# Sialic acid facilitates binding and cytotoxic activity of the pore-forming *Clostridium perfringens* NetF toxin to host cells

**DOI:** 10.1371/journal.pone.0206815

**Published:** 2018-11-07

**Authors:** Iman Mehdizadeh Gohari, Eric K. Brefo-Mensah, Michael Palmer, Patrick Boerlin, John F. Prescott

**Affiliations:** 1 Department of Pathobiology, University of Guelph, Guelph, Ontario, Canada; 2 Department of Chemistry, University of Waterloo, Waterloo, Ontario, Canada; Institut Pasteur, FRANCE

## Abstract

NetF-producing type A *Clostridium perfringens* is an important cause of canine and foal necrotizing enteritis. NetF, related to the β-sheet pore-forming Leukocidin/Hemolysin superfamily, is considered a major virulence factor for this disease. The main purpose of this work is to demonstrate the pore-forming activity of NetF and characterize the chemical nature of its binding site. Electron microscopy using recombinant NetF (rNetF) confirmed that NetF is able to oligomerize and form large pores in equine ovarian (EO) cell membranes and sheep red blood cells. These oligomeric pores appear to be about 4–6 nm in diameter, and the number of oligomer subunits to vary from 6 to 9. Sodium periodate treatment rendered EO cells non-susceptible to NetF, suggesting that NetF binding requires cell surface carbohydrates. NetF cytotoxicity was also inhibited by a lectin that binds sialic acid, by sialidase, and by free sialic acid in excess, all of which clearly implicate sialic acid-containing membrane carbohydrates in NetF binding and/or toxicity for EO cells. Binding of NetF to sheep red blood cells was not inhibited by the gangliosides GM1, GM2 and GM3, nor did the latter promote membrane permeabilization in liposomes, suggesting that they do not constitute the cellular receptors. In contrast, treatment of EO cells with different proteases reduced their susceptibility to NetF, suggesting that the NetF receptor is a sialic acid-containing glycoprotein.

## Introduction

*Clostridium perfringens* is a Gram-positive, spore-forming, obligate anaerobic bacterium [1,2]. This bacterium is well known for expressing a wide variety of toxins and enzymes that are directly related to its virulence [1,3,4]. The virulence factors involved in type A *C*. *perfringens*-associated enteric disease in foals and dogs have not been well characterized in the past, but our group has identified a toxin, designated NetF, which shows sequence homology to the Leukocidin/Hemolysin family of pore-forming toxins (PFTs), and which may be a key determinant of virulence in these animal species [5].

PFTs, which form the largest family of bacterial toxins, permeabilize cell membranes and induce ion imbalance [6]. According to the structural motifs involved in membrane insertion, they can be classified into α-PFTs and β-PFTs [7]. All known *C*. *perfringens* PFTs belong to the β- family [8].

These toxins share a common basic mode of action, which includes their secretion as soluble monomers into the extracellular environment of bacterial cells, followed by binding to specific host cell surface receptors. Binding of β-PFTs to the surface of target cells promotes a conformational change in each monomer and triggers the formation of an oligomer which then projects a β-barrel into the lipid bilayer of the cell membrane [9]. Clostridial β-PFTs belong to two main structural classes: a) the cholesterol-dependent cytolysins (CDCs) and b) the heptameric β-PFTs. The latter class contains two subfamilies, namely, the *S*. *aureus* α-toxin family and the aerolysin family [8]. The amino acid sequence of NetF shows significant homology with some important pore-forming toxins that belong to the former subfamily, including *C*. *perfringens* NetB toxin (48% similarity), *C*. *perfringens* delta-toxin (39% similarity), *C*. *perfringens* CPB toxin (34% similarity), and *S*. *aureus* alpha-toxin (30% similarity) [5]. The majority of CDCs recognize and bind to cholesterol as a cell membrane receptor. In contrast, the heptameric β-PFTs recognize different specific types of receptors [8] and thus differ in their activity towards different cell types and animals hosts. Among these toxins, those in the aerolysin family bind to specific receptors called glycosyl-phosphatidyl-inositol (GPI)-anchored proteins [8]. GPI-anchored proteins are widespread, and the aerolysin-like toxins thus are able to bind to a broad range of host cell types [10].

De and Olsen (2011) have shown that distantly related toxins from different organisms adopt a similar pore-forming architecture and that conservation of many of the amino acids residues seems to be essential for their binding and cytotoxicity [11]. The structure analysis of the *S*. *aureus* α-toxin family indicates a mushroom-shaped complex which contains three domains: β-sandwich, rim and stem domains. It has been reported that in this toxin family rim domain is involved in cell binding [8]. Savva *et al*. have identified the amino acid residues Trp-257 and Trp-262 on the rim domain of NetB are essential for binding of this toxin to cell surface receptor(s). They showed that the cytotoxicity and hemolytic activity of NetB toxin was markedly reduced by substitution of either position to alanine [12]. These residues are highly conserved in the α-hemolysin family as well as in NetF [5].

Confirming that NetF is actually a pore-forming toxin and identifying the cellular receptors involved in NetF-host cell interaction are important first steps in understanding its mode of action.

## Results

### Cytotoxic activity of rNetF

Recombinant NetF toxin was expressed without the signal peptide sequence and with an N-terminal his-tag (derived from the pET28a vector) that was removed using thrombin treatment. Both rNetF with and without N-terminal His-tag were evaluated for cytotoxic activity on EO cells. rNetF with His-tag was inactive on EO cells, whereas rNetF without the His-tag was highly toxic on the cells. This finding suggests that the N-terminal part of NetF plays a crucial role in its activity. Therefore, rNetF without a His-tag was used in all further experiments.

### Hemolytic activity of rNetF

**Fig 1:**
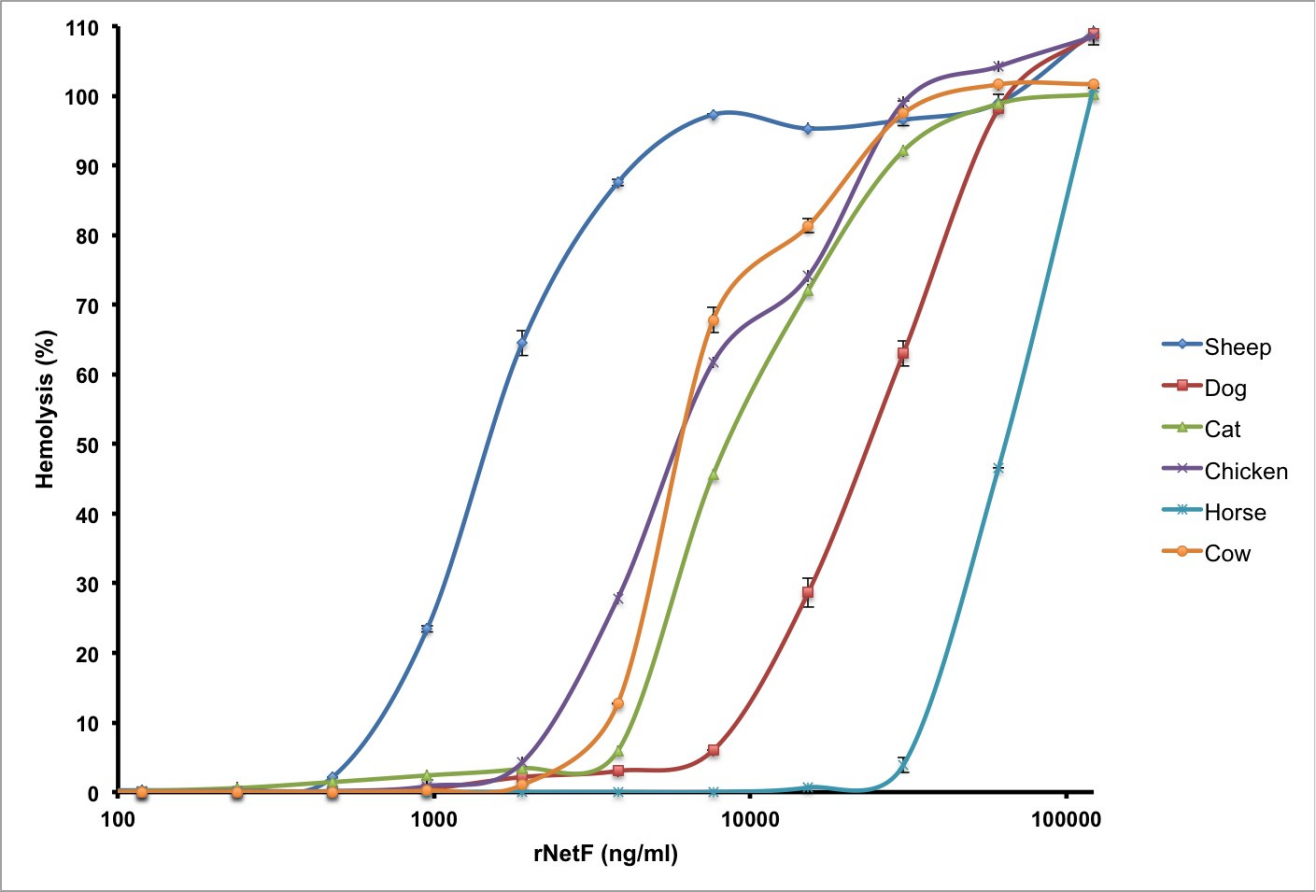
Quantitative analysis of hemolytic activity of rNetF. A two-fold serial dilution of rNetF was made in 96-well plates and incubated at 37°C for 1 h with RBCs (2.5% by volume) from six different species. Subsequently, the cells were pelleted and the supernatants were removed. Hemolysis was determined by measuring absorbance at 424 nm. PBS was used as the negative control for 0% hemolysis and 2% Triton X-100 as the positive control for 100% hemolysis. The values are averages of triplicate assays in three experiments, with error bars representing standard deviation.

rNetF was tested for hemolytic activity with RBCs from six different species (Fig 1). Among these, those from sheep were the most susceptible, with 50% hemolysis being observed at approximately 1.3 μg/ml, whereas those from horse were the least susceptible (50% hemolysis at 65 μg/ml). Consequently, sheep RBCs were used for all subsequent hemolysis studies. For biochemical studies, EO cells were used because of their markedly greater susceptibility to rNetF.

### Characterization of the NetF pore

**Fig 2:**
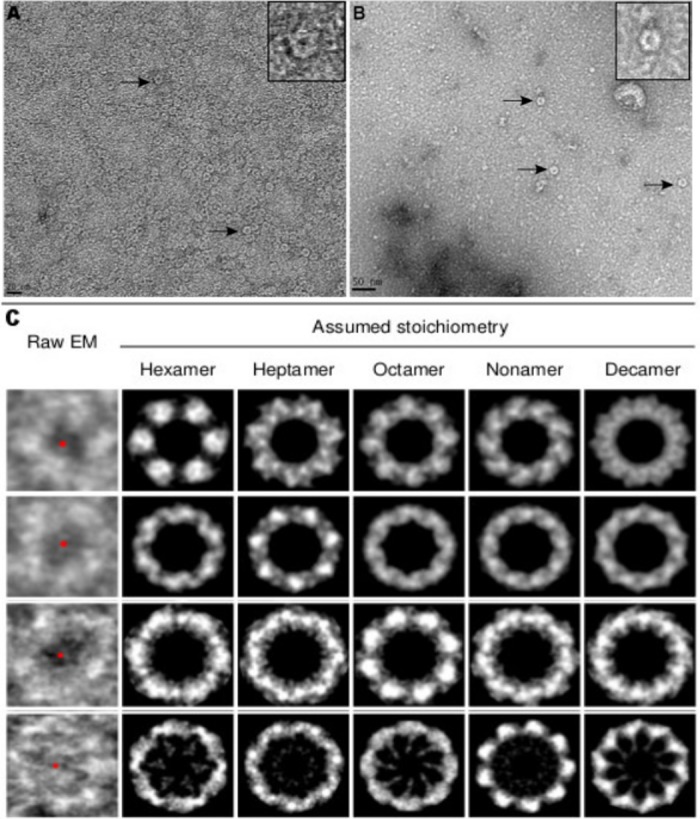
Transmission electron microscopy and symmetry determination of rNetF oligomers. **A-C.** The erythrocytes and EO cells were treated with rNetF toxin, and the lysed membranes were collected and washed several times by centrifugation. A and B) Representative views of negatively stained rNetF oligomers on sheep RBC (A; scale bar 20 nm) and EO cell (B; scale bar 50 nm) membranes. Arrows indicate individual membrane-bound NetF oligomers. The oligomeric structure of rNetF on sheep RBC and EO cells was shown on top right side of each figure. C) Top view of rNetF oligomers with different symmetry operations. All the EM pictures were acquired under the same conditions. For additional images and details of image analysis, see Supporting Information.

Since *netF* gene sequence analysis suggested that NetF might belong to the heptameric pore-forming toxin family, electron microscopy was used to observe the oligomerization and pore formation of this toxin on RBC and EO cell membranes. Ring-shaped NetF oligomers were observed on cell membranes (Fig 2A and 2B), confirming that NetF toxin is able to bind and oligomerize on cell membranes. In addition, the number of subunits in NetF oligomers on EO and RBC membranes was evaluated by image analysis (for details, see the Supporting Information). As can be seen in Fig 2C and S1C Fig, NetF oligomers may contain between 6 and 9 subunits; 6-fold symmetry (15%), 7-fold (49%), 8-(21%), and 9-(15%).

**Fig 3:**
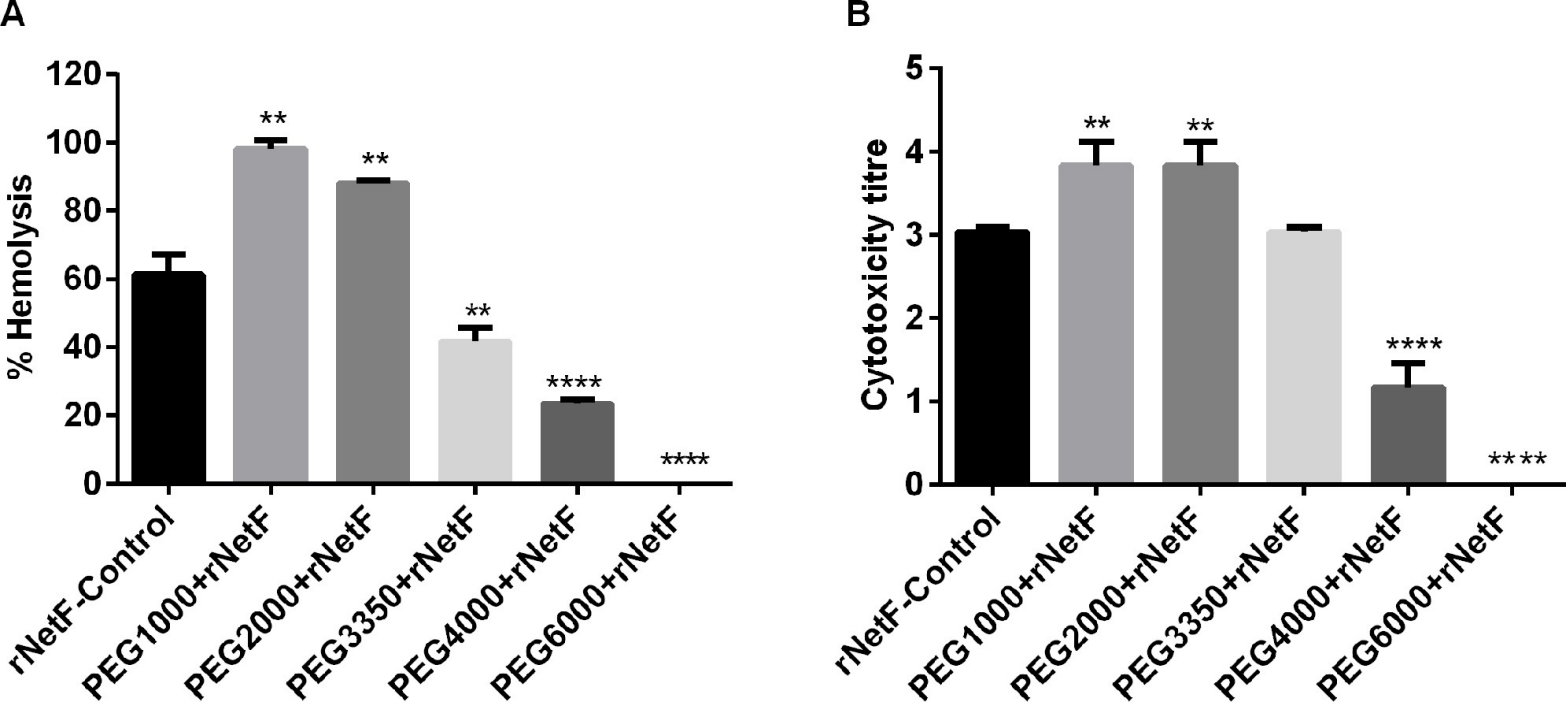
**A-B. Inhibition of rNetF-induced hemolysis and cytotoxicity by PEG.** A) Sheep RBCs (2.5% by volume) were incubated with EC_50_ concentration of rNetF (1.3 μg/ml of rNetF in the presence of 30 mM PEG (molar weight as indicated) at 37°C for 1 h. Hemolysis was determined by measuring absorbance at 424 nm. B) Confluent monolayer EO cells were incubated with EC_50_ concentration of rNetF (5 ng/ml) in the presence of 30 mM PEG (molar weight as indicated) at 37°C. Cytotoxicity was evaluated after 4 h. The values are averages of triplicate assays in three experiments, with error bars representing standard deviation. Results were considered statically significant from the control if p ≤ 0.01 (**) and p ≤ 0.0001 (****).

Hemolysis and cytotoxicity assays were performed in the presence and absence of polyethylengeglycols (PEGs) with various molecular weights to estimate the functional diameter of the pores on the membranes of both sheep red blood cells and EO cells. As shown in Fig 3A, PEG3350 induced a 32%, PEG4000 a 62% and PEG6000 a 100% inhibition of rNetF hemolytic activity with sheep RBCs, whereas PEG1000 and PEG2000 had no protective effect on rNetF-mediated hemolysis under the conditions tested. Similarly, rNetF-induced EO cytotoxicity was significantly inhibited by the larger molecular weight compounds (Fig 3B). The hydrodynamic radii of PEG4000 and PEG6000 have been estimated to be approximately 2 nm and 3 nm, respectively [13,14]. These results suggest that rNetF is able to form large pores on both EO and sheep RBC membranes with a functional diameter of approximately 4–6 nm. Interestingly, low molecular weight PEGs (≤ 2000 mM) increased the activity of rNetF in both experiments. PEG can be used for protein precipitation [15], and it seems possible that PEG promotes binding of proteins to each other not only in solution but also on membranes. Similar effects of PEG on hemolytic activity have been noted before [16]. Therefore, PEG may promote oligomerization and pore formation of NetF on sheep RBCs and EO cells membranes. In addition, TEM (transmission electron microscopy) image analysis using ImageJ revealed that the average size of pores generated by rNetF on cell membranes is approximately 5.4±1.08 nm, which closely matches the pore size estimation using PEG.

### Effect of NetF on artificial lipid membranes

**Fig 4:**
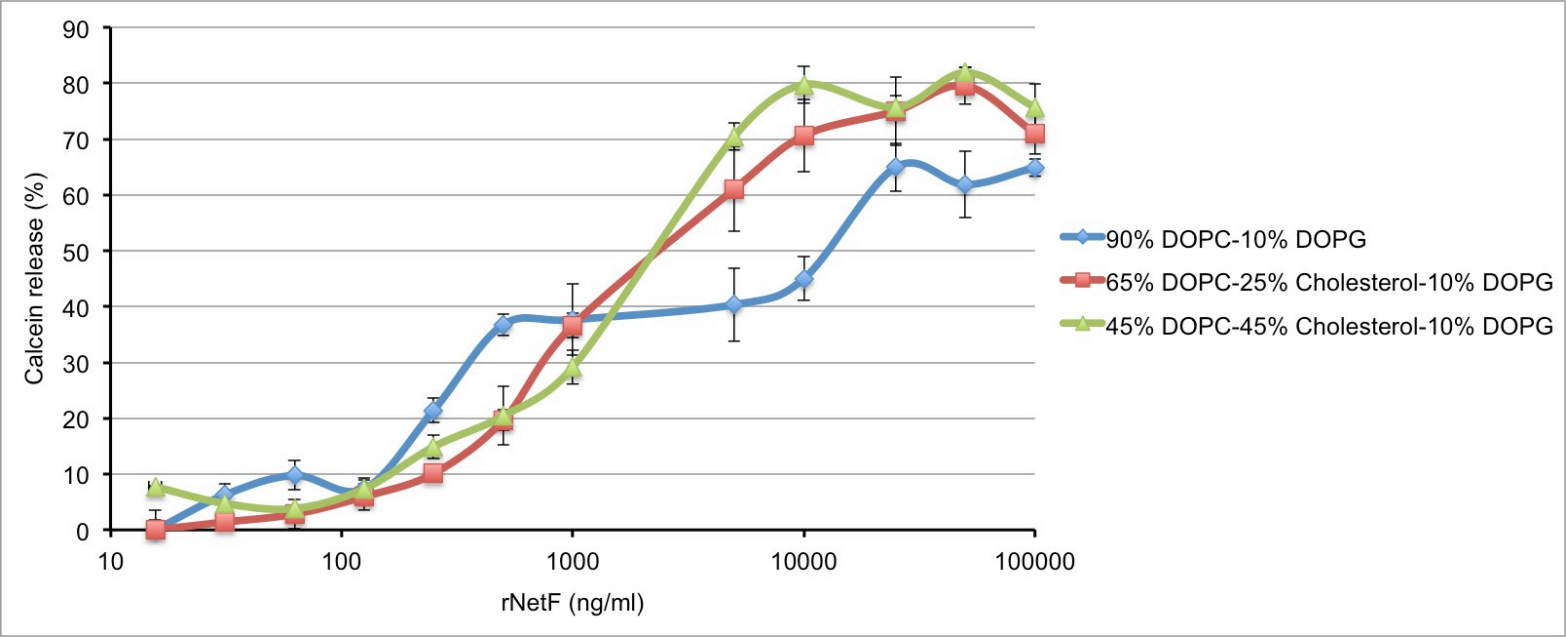
Permeabilization of liposomes by rNetF toxin. Liposomes of varying in lipid compositions (DOPC: DOPG: Cholesterol) and containing calcein were incubated with different concentrations of rNetF at 25°C for 1 h. The samples were then diluted and calcein fluorescence intensity was measured (excitation: 475 nm; emission: 516 nm). The extent of calcein release was quantified relative to a control sample that was solubilized with Triton X-100. The values are averages of triplicate assays in three experiments, with error bars representing standard deviation.

To analyse whether rNetF is able to form pores in artificial lipid membranes, liposomes composed of DOPC, DOPG and cholesterol in various molar proportions and containing the fluorophore calcein at self-quenching concentrations were incubated with various amounts of rNetF. On such liposomes, pore formation will cause the release of calcein, which can be detected as an increase in fluorescence intensity. As shown in Fig 4, rNetF dose-dependently released calcein from DOPC/DOPG liposomes. As also evident in Fig 4, inclusion of cholesterol in the liposomes promoted calcein release by rNetF at high concentration, but the effect was slight. No marked difference was observed in terms of calcein release between liposomes without or with 25 and 45 mol% cholesterol. While many other β-PFTs, including ones not belonging to the CDCs [12,17,18], are significantly affected by membrane cholesterol, no major effect was observed with NetF. Interestingly, it has been reported that NetB, the toxin most similar to NetF, can interact directly with cholesterol and that this association plays a crucial role in oligomerization and pore formation of NetB on liposome surfaces [12]. However, cell type specificity and different affinity of RBCs to this toxin is still questionable, because all cell surfaces contain cholesterol and even avian and mammalian RBCs express the same level of this lipid [19,20]. However, the possible effects of other membrane lipids on the activity of NetF remains to be further investigated.

### Biochemical characterization of molecules on EO cells required for activity of NetF toxin

**Fig 5:**
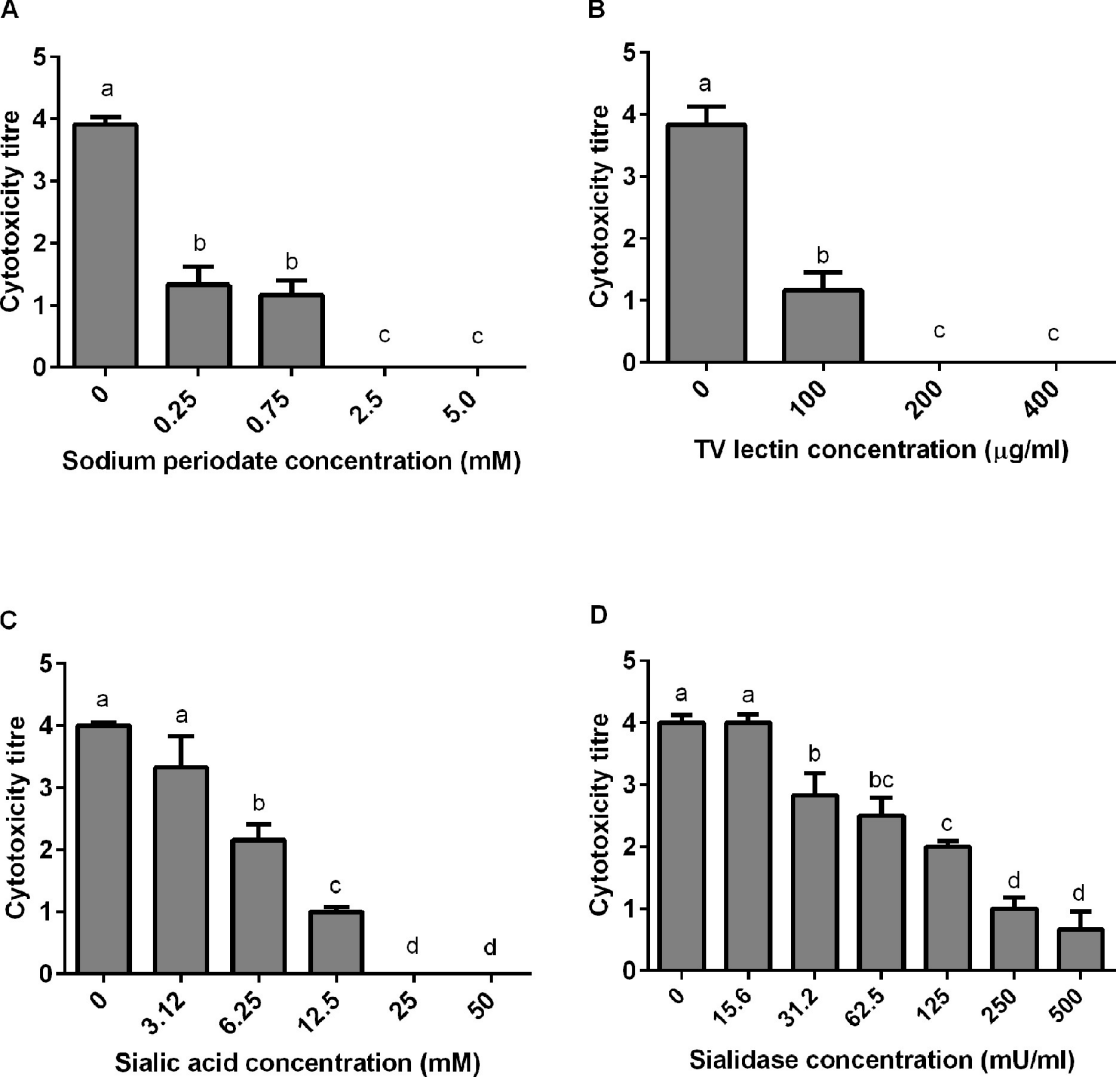
**A-D. Effect of sodium periodate, lectin, sialic acid and sialidase treatment on NetF binding molecules present on the surface of EO cells.** A) Cytotoxicity of NetF on EO cells pretreated with sodium periodate. Cells were incubated with various amounts of sodium periodate at 37°C for 20 min in order to destroy surface carbohydrates with vicinal OH groups. The cells were then washed and incubated with rNetF for 4 h before evaluating the toxicity score. B) Inhibition of NetF cytotoxicity to EO cell surface by TV lectin treatment. The EO cells were incubated with different concentrations of *Triticum vulgaris* lectin at 37°C for 20 min. The cells were then washed and incubated with rNetF for 4 h before evaluating the toxicity score. C) Inhibition of NetF cytotoxicity to EO cell by free sialic acid. rNetF was mixed with various amounts N-acetyl neuraminic acid (sialic acid) and incubated for 20 min at 37°C. This mixture was then transferred on EO cells and incubated for 4 h before evaluating the toxicity score. D) Effect of sialidase treatment on toxicity of NetF to EO cells. The EO cells were treated with different amounts of sialidase purified from *Arthrobacter ureafaciens* for 1 h at 37°C. The cells were then washed and incubated with rNetF toxin for 4 h before evaluating toxicity score. The values are averages of triplicate assays in three experiments, with error bars representing standard deviation. Bars which are marked by the same letter did not differ significantly (P > 0.05).

As stated earlier, toxins in the heptameric β-PFT family typically bind to cell surface glycoproteins or glycolipids [7]. To determine whether cell surface carbohydrates also mediate binding of NetF itself, EO cells were treated with sodium periodate. The susceptibility of EO cells to NetF was markedly diminished by this treatment and completely abolished at ≥ 2.5 mM sodium periodate (Fig 5A). Since sodium periodate is an oxidizing agent which destroys carbohydrates with vicinal hydroxyl groups, this suggested that the cell surface receptor or required factor for cytotoxic activity of NetF indeed is or contains a glycan moiety.

Lectins are proteins which recognize and bind to specific carbohydrates [21]. Treatment of EO cells with *Triticum vulgaris* (TV) lectin, which binds specifically to sialic acid and to N-acetyl-D-glucosamine, inhibited NetF cytotoxicity in a dose-dependent manner (Fig 5B). In contrast, EO cells treated with *Bandeiraea simplicifolia* (BS-I) lectin, which binds to α-galactose and N-acetyl-galactosamine, remained as susceptible to NetF as the control. These findings suggested that the cellular binding sites of NetF might contain either N-acetyl-D-glucosamine or sialic acid.

When either of these sugars were added to the assay as monosaccharides, only sialic acid inhibited NetF cytotoxicity on EO cells, completely abrogating it when used at 25 mM (Fig 5C), whereas N-acetyl-D-glucosamine had no effect.

To further confirm that sialic acid is important for cytotoxicity of NetF, we depleted cell surface sialic acid using sialidase enzymes and then measured the effect on NetF activity. *C*. *perfringens* sialidase did not decrease the susceptibility of EO cells, but *Arthrobacter ureafaciens* sialidase reduced the susceptibility of EO cells to NetF in a dose-dependent manner (Fig 5D). A possible reason for this discrepancy may be because bacterial neuraminidases differ in their activity toward sialoglycolipid substrates. For instance, sialidase from *C*. *perfringens* has 100-fold lower specificity for ganglioside GM1 than sialidase purified from *Arthrobacter ureafaciens* [22].

### Effect of gangliosides on permeabilization of sheep red blood cells or to liposomes

To test whether any of the monosialogangliosides GM1, GM2 or GM3 may be involved in binding of NetF toxin to cell membranes, each was tested for inhibition of hemolysis. *C*. *perfringens* delta-toxin, a pore-forming toxin that is homologous to NetF and a known ganglioside binder, was used as a positive control in this assay.

Pronounced inhibition of hemolysis indeed occurred with recombinant delta-toxin (rDelta), whereas inhibition was observed only at much higher concentrations with rNetF (S2 Fig). It was expected that the hemolytic activity of rDelta would be inhibited at lower ganglioside dosages because sheep RBCs are highly susceptible to this toxin and approximately a 130-fold lower concentration of rDelta compared with rNetF was needed to lyse 50% sheep RBCs.

Both toxins were also tested for permeabilization of liposomes into which each of the gangliosides had been separately incorporated. These liposomes did not show enhanced susceptibility to either rNetF or rDelta and thus did not provide conclusive evidence for or against a role of gangliosides in NetF activity (S3 and S4 Figs).

### Sialic acid containing-glycoprotein sensitizes EO cells to NetF

To define whether sialoproteins on the surface of EO cells are essential for attachment or toxicity of NetF toxin, EO cells were pre-treated with a panel of proteases that would destroy proteins (including sialoproteins) on the EO cell membranes.

**Fig 6:**
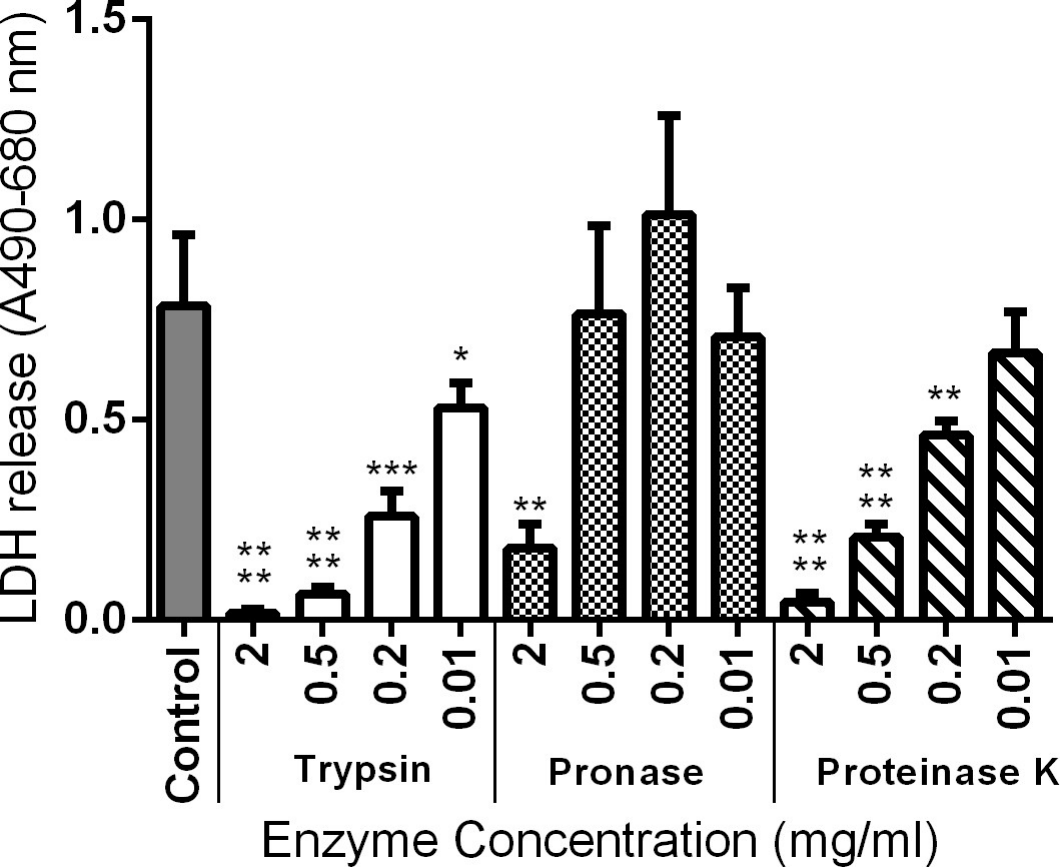
Effect of proteases treatment of intact EO cells on NetF cytotoxicity. Cells were treated with various concentration of proteases for 40 min. Following the washing steps, and protease inhibition, cells were incubated with rNetF at 37°C for 3 h. Subsequently, LDH release was used as indicator of cytotoxicity. The graph represents data from three replicates in three independent experiments (data are the means ± standard deviation). Results were considered statically significant from the control if p ≤ 0.05 (*), p ≤ 0.01 (**), p ≤ 0.001 (***), and p ≤ 0.0001 (****).

In this experiment, cell viability was also assessed after treatment with each enzyme, but before exposure to the toxin, and was found to not differ markedly from the untreated cells. As shown in Fig 6, protease treatment of EO cells reduced markedly the cytotoxicity of NetF toxin for EO cells in a dose-dependent manner, suggesting that the NetF receptor is a sialoprotein.

## Discussion

In the current study, we showed for the first time that NetF is indeed a pore-forming toxin (Fig 2A and 2B). Toxins in the *S*. *aureus* α-toxin family generally form heptameric pores on their targeted cell membranes [20,23,24]. For example, the molecular architecture analysis of NetB in detergent suggested that this toxin forms heptameric pores [12]. The appearance of the NetF oligomers observed here seems consistent with between 6 to 9 subunits (S1C Fig). Subunit stoichiometries from 6 to 8 have been described for homologous toxins. For instance, Czajkowsky *et al*. (1998) showed *S*. *aureus* α-toxin could form both hexamers and heptamers depending on the experimental conditions [25]. Moreover, *in vitro* studies on PV-leukocidin pores formed on RBCs and leukocytes have provided evidence for both hexamers and octamers [26–28]. Given this previously documented variability in the same toxin family, it is quite plausible that NetF could indeed form oligomers with variable subunit stoichiometry.

Based on the TEM and PEG studies, NetF pores appear to be larger than *S*. *aureus* α-toxin and NetB pores, which have an estimated size of 2.8 nm and 1.8 nm, respectively [29,30]. However, the size of NetF pores is similar to that of *C*. *perfringens* delta-toxin, which was estimated also to be approximately 5 nm [31]. In conclusion, while the approximate size of the NetF pores was determined, its ion selectivity–some related toxins such as *S*. *aureus* α-toxin and *C*. *perfringens* Delta toxin favour anions [23,31], others such as *C*. *perfringens* NetB and CPB toxin prefer cations [20,32]–and the details of its molecular architecture remain to be elucidated.

The hemolytic activity of NetF toxin is lower than that of the other members of this toxin family such as *S*. *aureus* α-toxin, *C*. *perfringens* delta- and NetB toxins. For instance, rabbit RBCs are highly sensitive to *S*. *aureus* α-toxin (maximum lysis at 15 ng/ml), whereas human RBCs are much less sensitive (no lysis at ~4 μg/ml) [33,34]. It has been shown that sheep RBCs are the most sensitive cells to *C*. *perfringens* delta-toxin, and its 50% hemolytic concentration is around 5–10 ng/ml [31]. Furthermore, Yan *et al*. (2013) showed that NetB toxin is more toxic on avian RBCs (duck [maximum lysis at ~5 ng/ml] and chicken [maximum lysis at ~2 μg/ml) than cells originating from other species (for example, 270 μg/ml of NetB is required for maximum lysis of horse RBCs) [20]. These findings suggest that the RBCs from different species do not express all the factors required for binding, conformational changes and oligomerization of β-pore-forming toxins.

The validity of using RBC as a system to identify the NetF receptor has not been established in this study, although it has been used in identification of receptor for α-toxin from *S*. *aureus* [35]. In addition, the liposome study in the absence of cholesterol suggests that NetF can form pores in the absence of receptors and therefore that it is likely that such pore formation also occurs in sheep RBC in the absence of specific binding to a receptor. The markedly lower toxicity of rNetF for sheep RBC compared to EO cells (~260 times lower), and the even greater difference for canine and equine RBCs, suggest that these RBC membranes do not have sufficient of the cell surface receptor or co-receptor which is required for binding and/or activity of NetF toxin, or indeed the receptor may be absent. Thus, the EO cell line was used in this study as a system to identify the receptor for NetF toxin, and multiple lines of evidence all point to a critical role of sialic acid in NetF binding to and toxicity for EO cells.

Gangliosides are glycosphingolipids with one or more sialic acid residues linked on the sugar chain [36] which are widely distributed in both human and animal tissues. Various types of gangliosides have been indicated as cellular receptor for bacterial toxins. For instance, ganglioside GM1 is the receptor for *E*. *coli* enterotoxin and cholera toxin [37,38]. Gangliosides G_D1b_ and G_T1b_ bind tetanus toxin [39,40]. Furthermore, ganglioside GM2 was identified as the cell surface receptor for *C*. *perfringens* delta-toxin [31,41]. Since both delta- and NetF toxins are members of the Leukocidin/Hemolysin superfamily, and since delta-toxin was found to bind to GM2, we hypothesized that the cell surface receptor for NetF might be a ganglioside, specifically GM2.

None of the gangliosides tested sensitized liposomes under the lipid compositions tested to NetF toxin and they only inhibited rNetF-mediated hemolysis weakly (S3 and S2A Figs). Since these gangliosides all contain a terminal sialic acid, it seems that the blocking of RBC lysis is most likely related to interaction of NetF with sialic acid and that these gangliosides do not serve as the specific cell surface receptor for NetF toxin. However, our findings with ganglioside-enriched liposomes as well as RBC inhibition assay do not allow us to rule out that gangliosides may contribute to the binding and possibly activity of NetF *in vivo*. Future work should examine the effect of these gangliosides on the toxicity of rNetF for EO cells.

Interestingly, the data also showed that rDelta toxin behaved similarly with each of the gangliosides (S2B Fig), in contrast to the work done by Manich *et al*. 2008 [31]. These workers showed that gangliosides GM1 (~5 μM) and preferentially GM2 (~2.5 μM) inhibited rDelta-mediated hemolysis, and that GM3 even at high concentration (10 μM) was unable to inhibit hemolytic activity of this toxin. However, in the present study, rDelta toxin did not show strong preference for GM2 in both experiments, and in addition, GM3 also inhibited the rDelta-induced hemolysis. This suggests that GM2 is probably is not the receptor for delta-toxin or that sialogangliosides may serve as an alternate receptor for function of delta-toxin on biological cell membranes.

When these data failed to support the hypothesis that gangliosides could act as a receptor for NetF toxin, we addressed whether a sialic acid containing glycoprotein would sensitize EO cells to NetF. Protease treatment made the EO cells resistant to NetF toxin (Fig 6), showing that the cell surface receptor or molecule required for toxicity of NetF to EO cells appears to be proteinaceous.

In conclusion, we demonstrate that NetF toxin is a pore-forming toxin and is able to form large, oligomeric pores on cell membranes. Although NetF probably has a common structure organization with other members of β-pore-forming toxins, its cellular receptor is still unclear and remains to be determined. We show that sialic acid and some yet unknown protein sensitize EO cells to NetF and we therefore conclude that the receptor is likely a sialoprotein.

## Materials and methods

### Production and purification of recombinant NetF

The full-length *netF* gene without signal peptide was amplified by PCR from strain JFP838 using the primers RecNetF-F (5`-CCGCGCTAGCAATTCCTTTCCTGAAAGTATTA-3`) and RecNetF-R (5`-CCGCCTCGAGTTAGTATATAAATTCTACAGTATGA-3`). The purified PCR product was cloned into the *Nhe*I-*Xho*I sites of pET-28a (Novagen, Gibbstown, NJ) to generate a recombinant protein fused with six N-terminal histidine residues from pET-28a. Subsequently, the resulting plasmid was transferred into *E*. *coli* BL21-Star (DE3) pLysS (Fisher Scientific, Hampton, NH). The *E*. *coli* BL21 strain containing recombinant plasmid was grown in LB broth supplemented with kanamycin (50 μg/ml) and chloramphenicol (34 μg/ml) at 37°C to an OD_600_ of 0.5. Then, the expression was induced by adding 1 mM isopropyl-β-D-thiogalactopyranoside (IPTG) at 37°C for 4 h. Purification of recombinant NetF (rNetF) from *E*. *coli* BL21 was conducted under non-denaturing conditions using Ni-NTA agarose following the manufacturer’s instructions (Qiagen, Mississauga, ON). The fractions containing the purified rNetF were collected and its buffer exchanged into 50mM Tris, pH 7.2, 200mM NaCl, 5% (vol/vol) glycerol using a Zeba spin desalting column (Fisher Scientific). Furthermore, the N-terminal 6-histidine motif was cleaved by thrombin enzyme (Novagen) according to the manufacturer’s instructions and then its buffer exchanged into Tris buffer as mentioned above.

### Cytotoxicity assay and cell line

An equine ovarian cell line (EO) (Dr. E. Nagy, University of Guelph; [5,42] was grown in complete Eagle’s Minimum Essential Medium (EMEM) (MEM/EBSS, L-glutamine 450 ml [GE Healthcare, Logan, Utah], 10% fetal bovine serum (FBS) [VWR International, Radnor, PA]). EO cells were incubated at 37°C and 5% CO_2_ in air. To test for cytotoxicity, EO cells were cultivated to almost 100% confluence in 96-well plates (Corning Inc., Corning, NY). For recombinant NetF cytotoxicity testing, filter-sterilized rNetF with and without the N-terminal his-tag was tested. Cytotoxicity was evaluated microscopically after 4 h and scored as previously described [5]. Briefly, confluent monolayers of the EO cell line with no evidence of toxicity were given a score of 0. Monolayers in which 25%, 50%, 75%, or 100% of cells were rounded or de-attached were given scores of 1+, 2+, 3+, and 4+, respectively.

### Hemolytic activity of rNetF

Hemolytic activity of rNetF was assessed using red blood cells (RBCs) from different animal species including horse, sheep, dog, cat, chicken, and cow. RBCs were washed three times in cold PBS buffer (20 mM NaH_2_PO_4_, 150 mM NaCl, pH 7.4) by centrifugation (3,000 × *g* at 4°C). The erythrocytes were then diluted in PBS to 2.5% (vol/vol). A two-fold serial dilution of rNetF (100 μl) was made in 96-well plates, and 100 μl of washed RBCs were then added to each well. The samples were incubated at 37°C for 1 h. Subsequently, the cells were pelleted and the supernatants were removed. Hemolysis was determined by measuring absorbance at 424 nm. PBS was used as the negative control for 0% hemolysis and 2% Triton X-100 as the positive control for 100% hemolysis.

### Demonstration of pore formation by NetF and estimation of pore size

To demonstrate pore formation by NetF, sheep RBC and EO cells were treated and lysed with 2 μg/ml and 20 ng/ml rNetF, respectively. After the cell lysis step, the membranes were collected by centrifugation (12,000 × *g* at 4°C). The membranes were then washed by centrifugation four times with cold PBS buffer. Subsequently, the final pellet was pipetted onto a Cu/Pd, 150 mesh, TEM grid with a formvar and carbon surface. The whole mount was stained with 2% uranyl acetate and viewed in a FEI F20 Tecnai TEM electron microscope (FEI, Hillsboro, Ore) at 120 kV with resolution of 0.25 nm.

An osmotic protection assay was performed to determine the size of pores generated by rNetF on sheep RBC and EO cells. These cells were prepared as described above for hemolysis and cytotoxicity assays and exposed to rNetF at a dosage that causes 50% of cell lysis (1.3 μg/ml for hemolysis assay and 5 ng/ml for cytotoxicity assay). The protective effect of polyethyleneglycol (PEG) with molecular weights ranging from 1000 to 6000 Da (each used separately at 30 mM final concentration) was quantified by the change in hemolytic or cytotoxic activity relative to the control sample without PEG. In both experiments, the percentage of osmotic protection was assessed based on hemolysis and cytotoxicity of rNetF in the absence of PEG. In addition, to validate the results of osmotic protection assay, ImageJ software [43] was also used to calculate the average pore sizes generated by rNetF of TEM images.

### EM structure of NetF pores

Details of EM image analysis are included with the Supporting Information.

### Activity of rNetF on liposomes with and without gangliosides

To prepare the liposomes, 1,2-dioleoyl-*sn-*glycero-3-phosphocholine (DOPC), cholesterol, and 1,2-Dioleoyl-*sn*-glycero-3-phosphoglycerol (DOPG) (Avanti Polar Lipids, Alabaster, AL) were dissolved in chloroform and mixed in various molar ratios. Three liposome formulations were prepared: DOPC: DOPG (90%: 10% molar ratio), DOPC: cholesterol: DOPG (65%: 25%: 10% molar ratio) and DOPC: cholesterol: DOPG (45%: 45%: 10% molar ratio). Where applicable, the gangliosides GM1, GM2, and GM3 (Sigma, St. Louis, MO; dissolved in chloroform: ethanol 1/1) were added at 2% of the total lipids. The lipid solutions were dried down under a stream of nitrogen in a round bottom flask for 5–10 min and further dried using a vacuum desiccator for 3 h. The dried lipids were hydrated with 3.0 ml of Hepes-buffered saline (HBS; 10 mM HEPES, 150 mM NaCl, pH 7.4) containing 50 mM calcein (Sigma) at room temperature by vortexing for 20 min to a final total lipid concentration of 3 mg/ml. The resulting suspension of multilamellar liposomes was converted to unilamellar liposomes using a liposome extruder (Northern Lipids, Vancouver, BC) by extruding 15 times through a 100-nm polycarbonate membrane filter (GE Healthcare). Subsequently, non-encapsulated calcein dye was removed by gel filtration using a Bio-Gel A-1.5m Gel (Bio-Rad Laboratories, Hercules, CA) column which was pre-equilibrated with HBS. The liposomes with encapsulated calcein (approximately 34 μg) were mixed with different concentrations rNetF in HBS buffer and incubated at 25°C for 1 h. The samples were then diluted with HBS buffer and calcein fluorescence intensity was measured immediately using a QuantaMaster 4 spectrofluorometer (PTI, London, ON) with an excitation and emission wavelength of 475 and 516 nm, respectively. Calcein release percentage was calculated with the following formula:

$$Calcein\;release\;\left(\%\right)=100\;*\;\left(F_{sample}–F_{0}\right)/\left(F_{Triton}–F_{0}\right)$$

where F_0_ is the fluorescence of a liposome control without toxin, and F_Triton_ is that of a liposome sample solubilized with at a final concentration of 0.01%.

For this assay, rDelta toxin of *C*. *perfringens*, prepared as previously described [32], was used as a positive control.

### Sodium periodate, lectin, sugar and sialidase treatment of cells

Periodate treatment was performed to determine whether the NetF receptor or required co-factor for its toxicity is a carbohydrate or contains a glycan moiety. For this purpose, confluent EO cells to be treated with sodium periodate (Sigma) were washed once with pre-warmed PBS (pH 7.4). The EO cells were incubated with various concentrations of sodium periodate in sterile MilliQ H_2_O (0, 0.25, 0.75, 2.5, 5.0 mM) at 37°C for 20 min. Then, the cells were rinsed twice with PBS and incubated with rNetF (20 ng/ml) for 4 h before evaluating cytotoxicity.

To confirm the importance of carbohydrate in the NetF receptor, and to further characterize its possible nature, carbohydrate-binding lectins were used, including *Triticum vulgaris* (Sigma) (TV) and *Bandeiraea simplicifolia* (Sigma) (BS-I) lectins, which are specific for sialic acid/N-acetyl-D-glucosamine and α-galactose/N-acetylgalactosamine, respectively. In this assay, inhibition of NetF cytotoxicity was assessed with various concentrations (0, 100, 200, 400 μg/ml [H_2_O]) of BS-I and TV lectins, which were added to EO cells after washing with pre-warmed PBS. After 2 h incubation at 37°C, the cells were washed twice and then rNetF (20 ng/ml) was added to the treated cells and incubated for 4 h before evaluating cytotoxicity.

To further examine the carbohydrate nature of the receptor or co-factor required for activity of NetF suggested by the lectin studies, rNetF was mixed (vol/vol) with different amounts (3.1, 6.3, 12.5, 25, 50 mM) of N-acetyl-D-glucosamine (Sigma) or N-acetyl neuraminic acid (Sigma) and incubated for 20 min at 37°C. Subsequently, each mixture was mixed (vol/vol) with complete EMEM medium and added to EO cells. Sterile MilliQ H_2_O was used to dilute the carbohydrates and also as a negative control. Subsequently, cytotoxicity was evaluated after 4 h as described above.

Sialidase treatment was used to confirm that sialic acid was the glycan moiety essential for interaction or function NetF on EO cell line. In this assay, the cells were initially rinsed with pre-warmed PBS and then incubated in Hanks’ Balanced Salt Solution (HBSS; pH 5.85) containing various concentrations of either *C*. *perfringens* sialidase or *Arthrobacter ureafaciens* sialidase (Sigma) (15.6, 31.2, 62.5, 125, 250, 500 mU/ml [one unit is the enzyme activity that releases 1μmol N-acetyl neuraminic acid in 1 min at 25°C] for 1 h at 37°C. The cells were then washed twice with PBS. Subsequently, rNetF (20 ng/ml) was added to the treated cells and incubated for 4 h before evaluating cytotoxicity. Cells treated with HBSS were used as a negative control.

To examine whether a ganglioside might be the NetF receptor, each of the gangliosides GM1, GM2, and GM3 was tested for inhibition of hemolysis. The gangliosides were dissolved in dimethyl sulfoxide to 10^−3^ M and then diluted in PBS. rNetF (1.3 μg/ml) was supplemented with various amounts (see Results section) of each ganglioside and then incubated at 37°C for 20 min. Subsequently, 100 μl of 2.5% sheep RBC was added to each mixture and hemolytic activity was measured as described above. In this assay, dimethyl sulfoxide diluted in PBS was used as a control.

### Treatment with proteases

Sialic acid residues occur in the glycan moieties of both glycoproteins and glycolipids. To clarify whether cell surface glycoproteins are important for interaction of NetF with EO cells, a series of experiments using different proteases was initially performed. This assay was carried out using cell suspensions since protease treatment would cause cells to detach from the substrate. Therefore, EO cells were detached from culture flasks by 10 mM EDTA in HBSS buffer before further testing.

After washing the cells twice with HBSS buffer, approximately 2 x 10^4^ cells were treated with proteases in HBSS at pH 7.0 for 40 min at 25°C. The enzymes used in this experiment were as follows: Proteases: Trypsin from bovine pancreas (Sigma), Pronase from *Streptomyces griseus* (Sigma), and Proteinase K from *Pichia pastoris* (Sigma), each at concentrations of 2, 0.5, 0.2, and 0.01 mg/ml. After incubation, cells were extensively washed five times by centrifugation with EMEM medium containing 10% FBS to remove enzymes. To minimize the activity of any residual proteases, the washing medium was supplemented with protease inhibitor cocktail (Sigma) which contains Pepstatin A, Leupeptin, E-64, Bestatin, Aprotinin, and AEBSF. Following the washes, cells were incubated with rNetF (20 ng/ml) at 37°C for 3 h. The protease inhibitor cocktail was also added to the toxin sample to protect it from any residual protease activity. For this experiment, lactate dehydrogenase release (LDH) in the supernatant was used as indicator of cytotoxicity. The Pierce LDH cytotoxicity assay kit (Fisher Scientific) was used to measure LDH release. Cells treated with enzyme buffer only were used as controls in this study. In addition, the LDH release after each enzyme treatment (prior toxin treatment) was also measured to assess cells viability after treatment.

### Statistical analysis

One-way analysis of variance (ANOVA) was used for statistical analyses. Because the ANOVA approach assumes the data to follow a Gaussian or normal distribution, which cannot be judged or tested from 3 replicates only, the non-parametric Kruskal-Wallis Rank Sum test was applied as an alternative method as well. All the experiments presented in the current study were repeated three times independently and the results were reproducible. Each independent experiment contained three technical replicates.

## Supporting information

S1 Fig Figs A-D. NetF oligomer subunit stoichiometry: methods and additional data.
(PDF)

S2 Fig Inhibition of rNetF (A) and rDelta (B)-mediated hemolysis by gangliosides.
(PDF)

S3 Fig rNetF-induced calcein release from liposomes containing gangliosides. (PDF)

S4 Fig rDelta induced calcein release from liposome containing GM2. (PDF)
