# Supplementary material for: Sialic acid facilitates binding and cytotoxic activity of the pore-forming Clostridium perfringens NetF toxin to host cells
Source: PLoS One. 2018 Nov 7;13(11):e0206815. doi: 10.1371/journal.pone.0206815 (PMC6221314; doi:10.1371/journal.pone.0206815)
Supplement: S1 Fig — Figs A-D. NetF oligomer subunit stoichiometry: methods and additional data. (PDF) [file pone.0206815.s001.pdf]

NetF oligomer subunit stoichiometry: methods and additional data

1.1 Rationale and technical approach

To determine the subunit stoichiometry of membrane-bound NetF oligomers from electron microscopy (EM) pictures, we decided to examine individual oligomers rather than to construct averaged overlays from multiple oligomers. We did so because subunit stoichiometries from 6 to 8 have been described for homologous toxins, and with at least one such toxin (*Staphylococcus aureus* alpha toxin), the stoichiometry has been found to vary depending on the experimental conditions [1]. If such heterogeneity were to occur with NetF—and inspection of the EM pictures did suggest this possibility—the averaging of multiple oligomers would likely not be informative.

To analyze each oligomer, it was first cropped from the raw EM picture and then scaled up (with antialiasing) by a factor of 4; the latter was done to reduce the effects of rounding errors during the subsequent processing stages. The image was then overlaid with itself at multiple angles; for example, to see whether the oligomer might be a hexamer, it was superimposed at 60, 120, 180, 240, and 300 degrees. More generally, to test the assumption of n subunits, the angles were $^{360}/_{n}$, 2 x $^{360}/_{n}$, . . . , (n-1) x $^{360}/_{n}$.

The approach is illustrated in S1A Fig. The underlying assumption is that the subunits in a given oligomer are placed at fixed angles along a circle, but the brightness of each subunit can vary randomly. Then, if we superimpose the image with itself at the

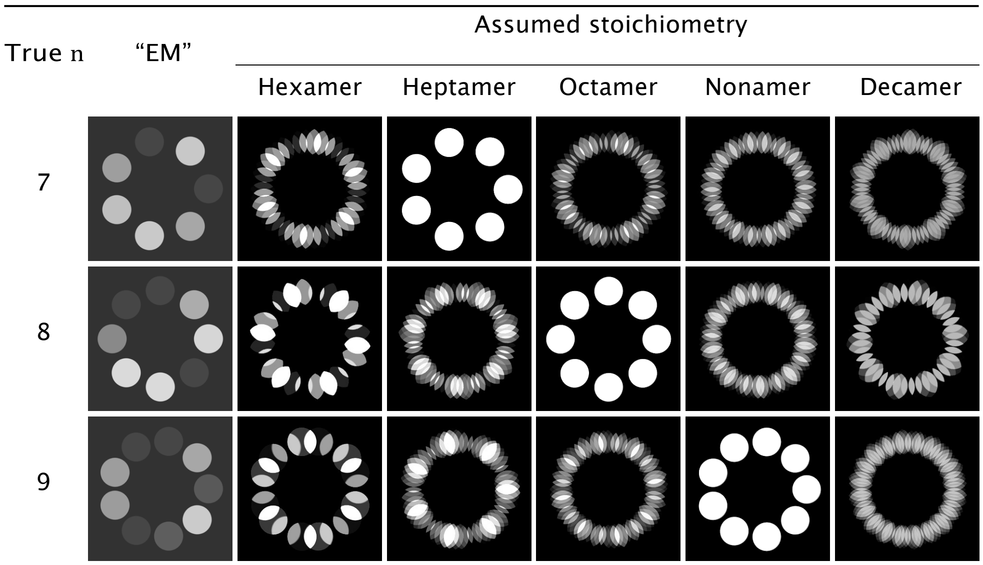

S1A Fig: Illustration of the rotational overlay method for determining oligomer subunit stoichiometry (n). The column labeled “EM” shows “oligomers” with regular positioning but uneven brightness of subunits. Rotational overlays give the sharpest contrast if they match the true n. Note, however, that spurious signals occur, e.g. the “true” nonamer also gives fairly bright spots in the assumed heptamer slot.

correct angles that match the oligomer’s rotational symmetry, all subunits should fall into register to produce the sharpest possible contrast along the circle.

While with simulated oligomers the center point is of course known, that is not the case with real ones. Since the distribution of intensity in these cropped images is quite uneven, we did not attempt to calculate the center from those intensity values; instead, we varied the center location systematically and visually judged how well each position fit the oligomer in question. The approach is illustrated in S1B Fig.

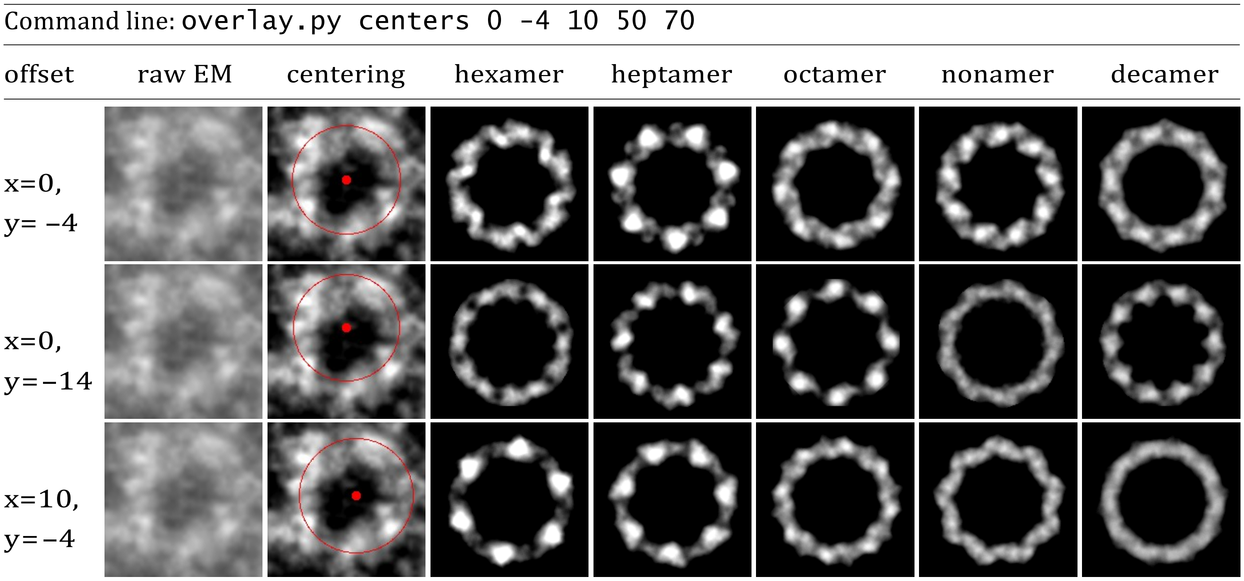

S1B Fig: Example rotational overlays with manual centering. See text for details.

The program[1] was given the x and y coordinates of the center of rotation, as well as an interval of variation around it to explore.[2] Applying the variation interval in positive and negative direction to the center coordinates, singly or together, gives nine combinations overall, three of which are shown in this example. In the topmost row in S1B Fig, the center of rotation was offset from that of the picture by 0 pixels horizontally and −4 pixels vertically. In the other two rows in S1B Fig, the additional offset of 10 pixels (a rather large value, chosen for the sake of illustration) was applied in vertical and horizontal direction, respectively.

In each row, the *centering* column shows a contrast-enhanced copy of the raw image, marked with the tentative center of rotation, as well as with the estimated oligomer radius.[3] With the adjustment used in the topmost row, the image was deemed satisfactorily centered (this will be easier to judge when zooming in). Note how shifting the

[1] The program used to carry out this analysis was implemented in Python; the code listing (with explanatory comments embedded) is given in Section 1.3.

[2] x, y, and the variation interval are the first three numbers on the command line given in S1B Fig. The final two parameters control the contrast enhancement of the overlaid figures.

[3] The radius is calculated as the average distance of the brightest 10% of all pixels in the picture, which will mostly fall onto the oligomer ring, from the chosen center of rotation.

center affects the appearance of the overlays: the topmost row is very suggestive of heptamers, while the other two are rather more ambiguous.

1.2 Results

For all of the oligomers evaluated, centering was performed first, and the radius estimated by the program relative to the hand-picked center was recorded. The first and second choice of subunit stoichiometry was visually determined. Some oligomers (about $^1/_8$ of all that were initially processed) did not yield any visually preferable stoichiometry and were excluded at this stage. The results for the remaining ones are shown in S1C Fig.

S1C Fig: Rotational overlays and interpretations of individual oligomer images. The first choice is the stoichiometry we considered most likely, the second is the one we considered the next most likely.

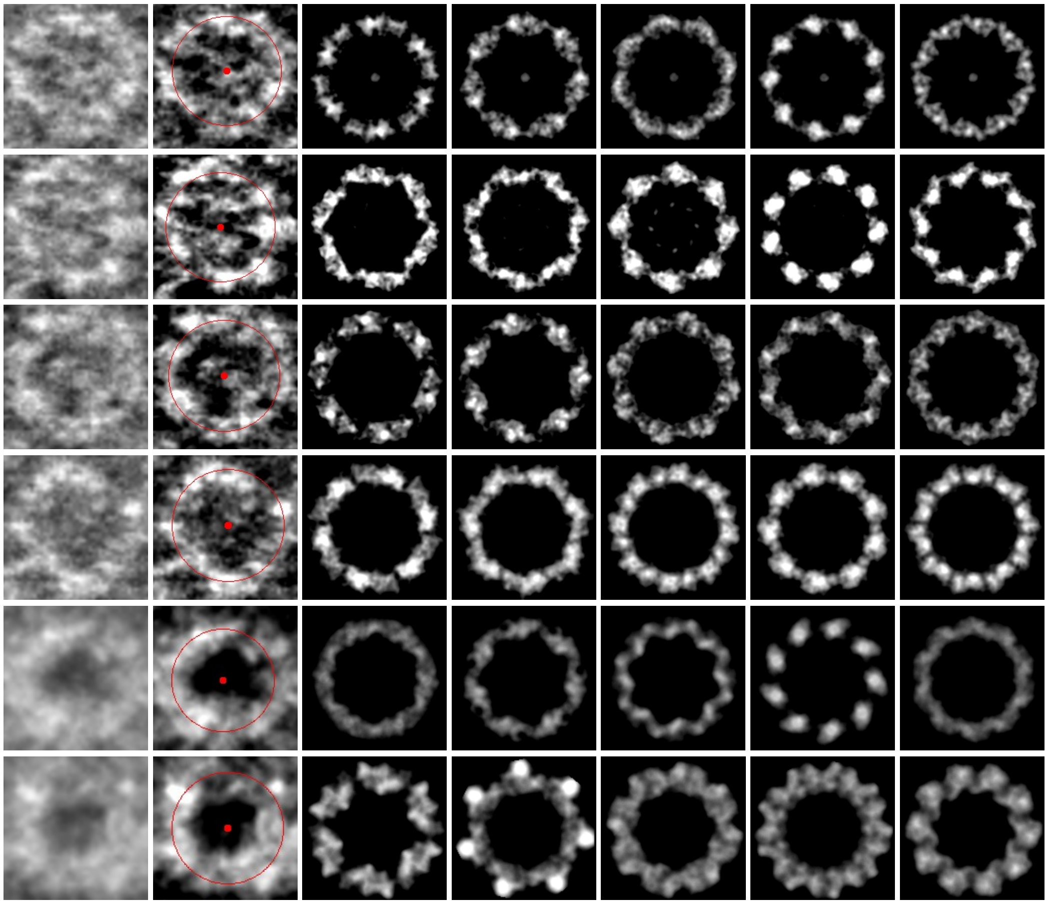

raw EM	centering	assumed stoichiometry					choice	
		hexamer	heptamer	octamer	nonamer	decamer	first	second
							9	8
							9	8
							7	9
							9	7
							9	7
							7	6

raw EM	centering	assumed stoichiometry					choice	
		hexamer	heptamer	octamer	nonamer	decamer	first	second

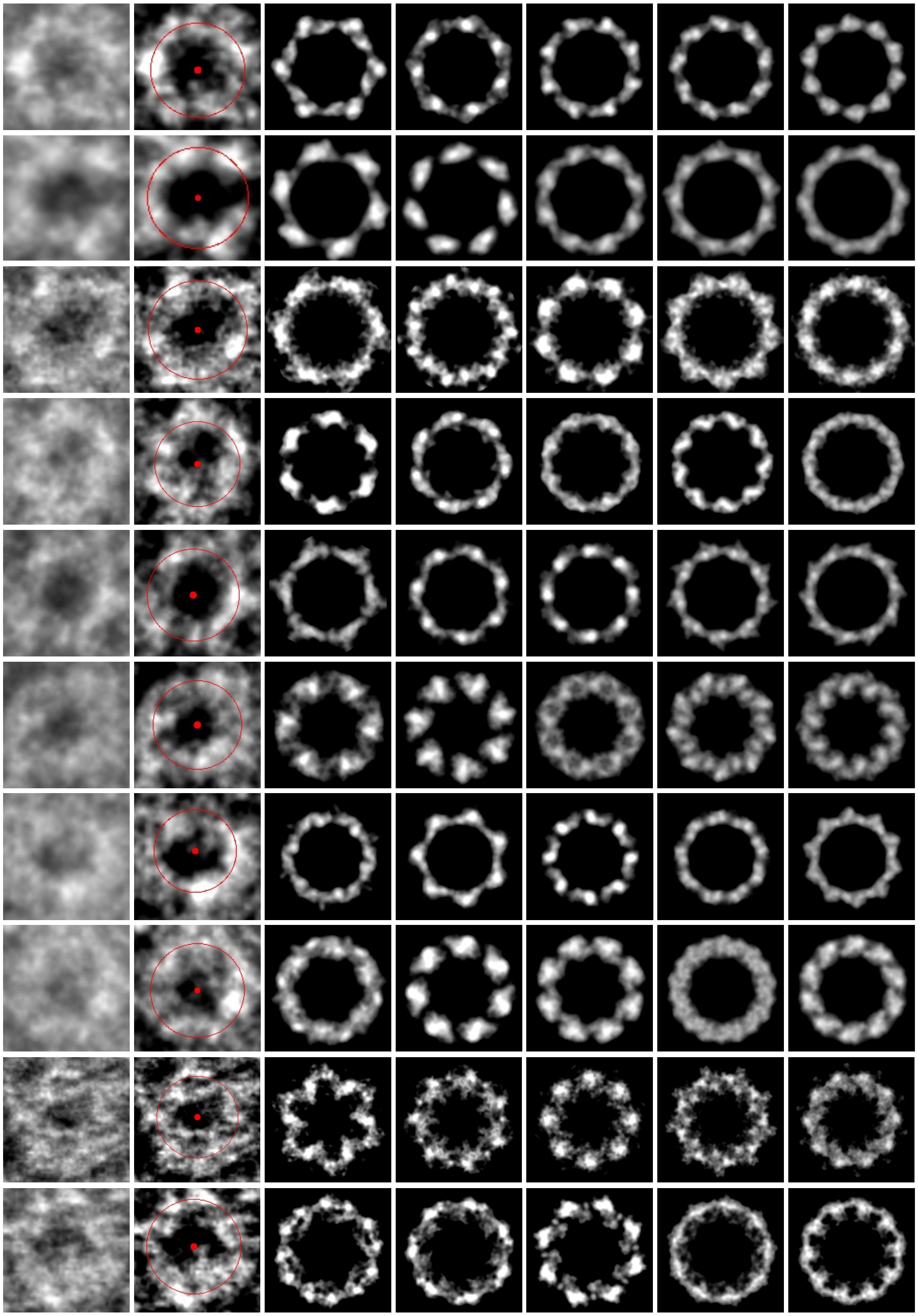

first	second
7	9
7	6
8	6
6	7
8	7
7	6
7	8
7	8
8	7
8	7

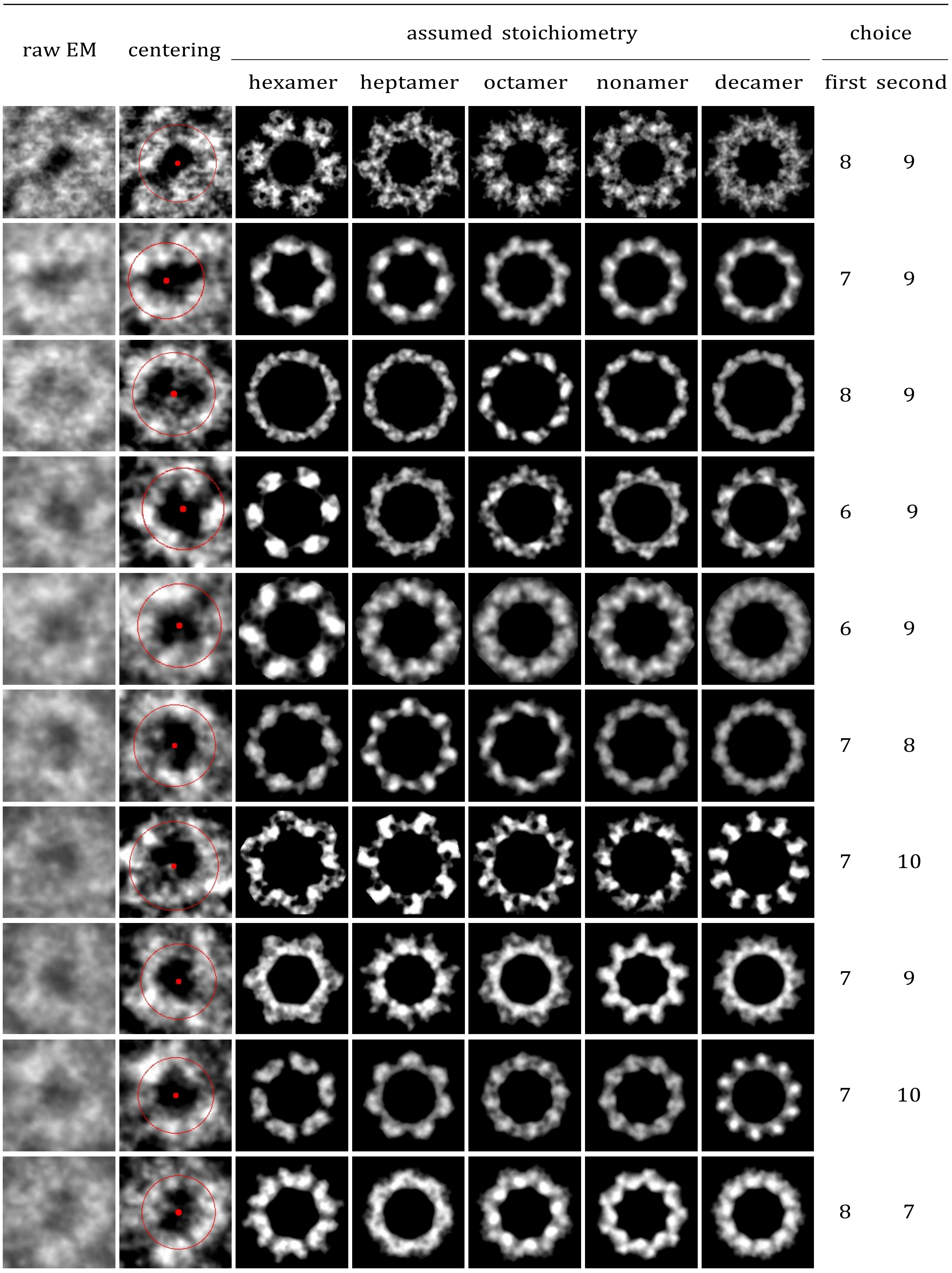
raw EM
centering
assumed stoichiometry
hexamer
heptamer
octamer
nonamer
decamer
choice
first
second
8 9
7 9
8 9
6 9
6 9
7 8
7 10
7 9
7 10
8 7

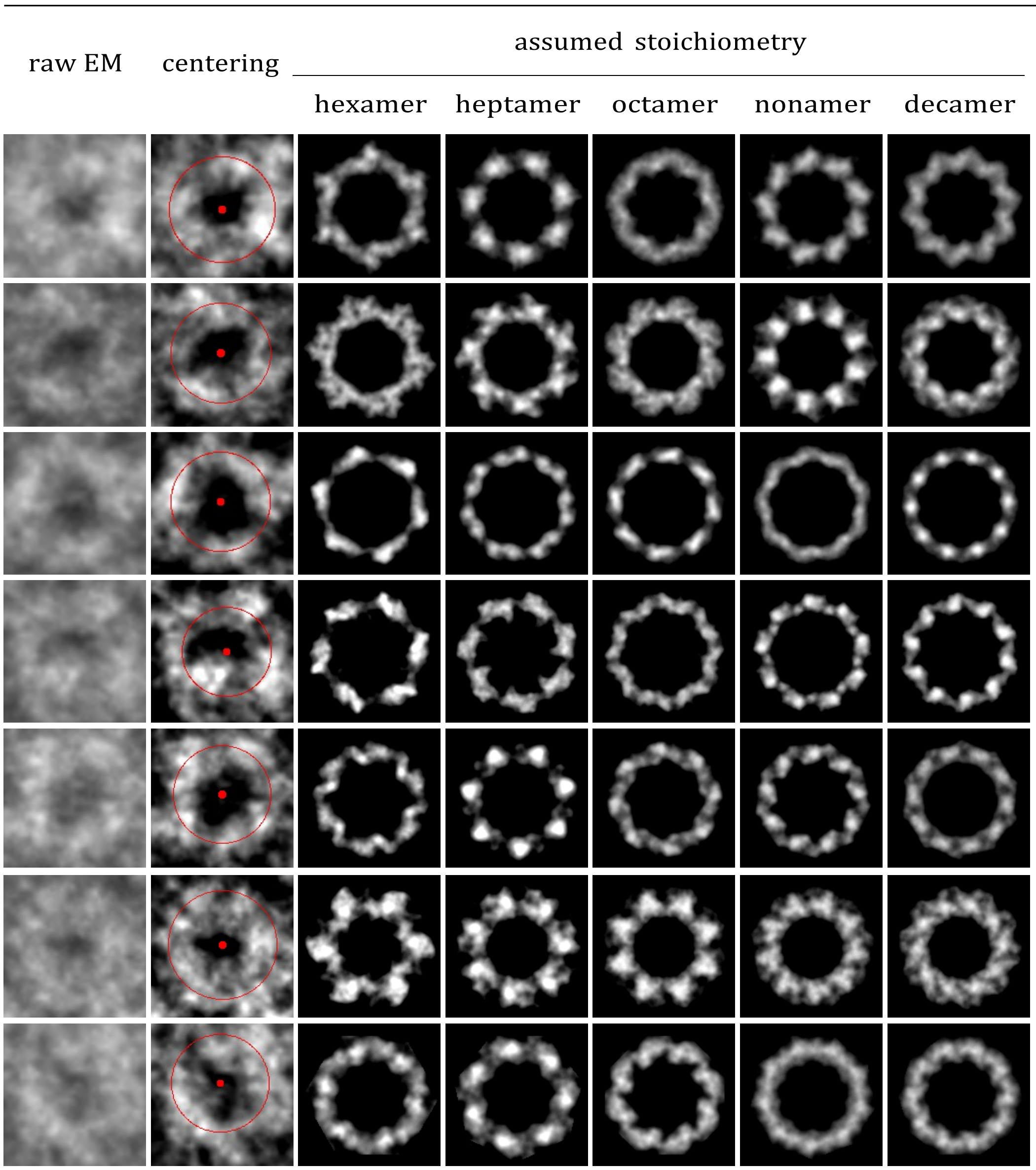

choice	
first	second
7	9
9	7
6	8
6	7
7	8
7	8
7	8

As detailed in the figure, we identified likely specimens of all subunit stoichiometries ranging from 6 to 9. Since most toxins with homology for NetF have been assigned defined, unique stoichiometries, one might doubt if this can be true. If so, one should expect a systematic correlation of the stoichiometry with the radius of the oligomer.

In S1D Fig, the estimated oligomer radius—as represented by the red circles in Table 1—is plotted as a function of the number of subunits. In panel A, all first-choice values for n were used. The radius indeed does increase with the inferred number of subunits, but the correlation is not particularly close. If we remove the outliers indicated by solid points in panel A (6 out of 33 total) as likely errors, the correlation coefficient increases markedly. The equation of the trend line for this reduced set is y = 7.9x— 0.9.
In other words, it passes within one pixel through the origin and thus gives the expected direct proportionality of radius and subunit number.

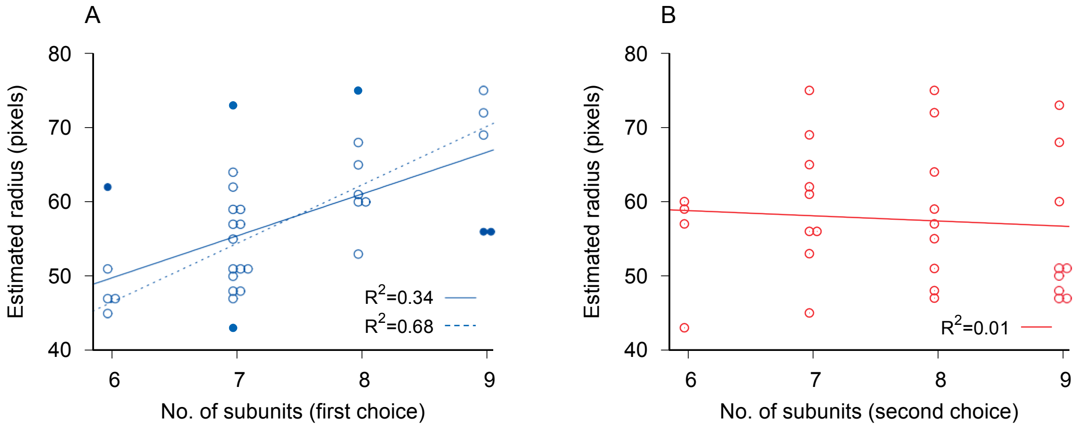

S1D Fig: Estimated oligomer radius *vs.* inferred number of oligomer subunits, for the first choice (A) and the second choice (B) of subunit stoichiometry (cf. S1C Fig). In A, linear trend lines are given with all points included (solid line) or with outliers (filled circles) excluded (dashed line).

Considering that the algorithm used for estimating the oligomer radius is fairly simplistic and susceptible to noise, the observed correlation suggests that after exclusion of outliers most of the remaining oligomers have been correctly assigned. In contrast, no correlation exists between the stoichiometry chosen (at times somewhat arbitrarily) as the second most likely and the estimated radius. Overall, these findings support the notion that NetF oligomers can have between 6 and 9 subunits (and possibly numbers outside this range that were not detected here).

1.3 Program code

```
'''
This program was used to generate the rotated EM overlay figures of NetF pores
in Mehdizadeh Gohari et al. (2018)

The program is written for Python 2 and requires the Python Imaging Library

https://pypi.python.org/pypi/Pillow

The PIL version should match that of the Python interpreter exactly, or
it may not work.

The program is invoked from the command line. It expects the clipped image
to be analyzed to reside in a subfolder, say, image1, and be named 'test.png'.

/.../overlay.py
/.../image1/test.png

It also expects a number of positional parameters:
```

```
python overlay.py <folder> <x offset> <y offset> <variation> <t_black> <t_white>

Example: python overlay.py image1 0 0 5 50 70

folder:     image1 in our example

x offset:   Horizontal offset of the center of rotation, in pixels, relative
            to the center of the image

y offset:   Vertical offset of the center of rotation. Negative values will
            shift the center up, positive will shift it down.

variation:  For each assumed subunit stochiometry (6 to 10 by default), the
            program will generate 9 different overlays, with the center of
            rotation offset by -variation, 0, and +variation, in both x and  y

            These overlays will be named "averaged[n][xy].png", where [n]
            is the assumed stoichiometry, and [xy] is one of ll, lz, lh,  zl,
            zz, zh, hl, hz, hh, depending on whether the offset in x or y was
            negative (l), zero (z) or positive (h).

            For each of the 9 variant centers, the program will also generate
            a corresponding "marked[xy].png" file in which the center and the
            estimated radius of the oligomer are marked.

            If a value of 0 is passed for the variation, then only one overlay
            file (named averaged[n]zz.png) is generated.

            I have a LaTeX file to arrange all of these generated files into
            a nice table, which I will send to you upon request. Alternatively,
            you could just use PIL to do the same thing.

t_black:    Used for contrast enhancement. On a scale of 0 (black) to 100 (white),
            all values below t_black will be converted to black, and all values
            above ...

t_white     ... will be converted to white. Values between both thresholds will
            be scaled linearly. E.g. with t_black=50 and t_white=70, native
            brightness of 55 will be converted to 25, 60 to 50, and so  on.

Written by: Michael Palmer (mpalmer@uwaterloo.ca); copyright: public domain.
'''

from PIL import Image, ImageDraw, ImageEnhance
import math, sys

# some global settings that I had no need to change, but you might

n_from = 6                    # lowest assumed stoichiometry
n_to = 10                     # highest assumed stoichiometry
```

```
original_file = 'test.png'  # file name of original (cropped) EM image


def matrix_rotate(point, angle,offset=(0,0)):
    '''
    rotate a point by an angle, with optional offset

    x' = x cos(a) - y sin(a)
    y' = x sin(a) + y cos(a)

    PIL uses a top-left origin coordinate system. To deal
    with this, we invert y twice and hope for the best.
    '''
    a = angle * math.pi/180
    cos = math.cos(a)
    sin = math.sin(a)

    x, y = point
    x_offset, y_offset = offset

    y = -y

    xt = x * cos - y * sin
    yt = x * sin + y * cos

    xt -= x_offset
    yt += y_offset

    return int(xt), int(-yt)


def rotation_offset(img, angle):
    '''
    calculate the x and y offset resulting from the enlargement
    of the canvas that results from rotating an image.
    '''
    width, height = img.size
    xc, yc = [], []

    for point in ((0, 0), (width, 0), (0, height), (width, height)):
        trafo = matrix_rotate(point, angle)
        xt, yt = trafo
        xc.append(xt)
        yc.append(yt)

    return min(xc), min(yc)


def pivot_offset(img, pivot, angle):
    '''
```

```
    calculate the offset of the pivot point relative
    to the image's (0,0) coordinate, after rotating
    the image about the origin by the angle.
    '''
    ro = rotation_offset(img, angle)
    pivot_x, pivot_y = matrix_rotate(pivot, angle, ro)
    return pivot_x, pivot_y


def draw_pivot(canvas, img, angle=0, pivot=(0,0), placement=(0,0)):
    '''
    draw img on canvas, rotated by angle, and centering
    pivot point on placement coordinates.
    '''
    x_pi, y_pi = pivot_offset(img, pivot, angle)
    x_pl, y_pl = placement

    x_img = x_pl - x_pi
    y_img = y_pl - y_pi

    img_r = img.rotate(angle, resample=Image.BICUBIC, expand=1)
    canvas.paste(img_r, (x_img,y_img))


def apply_contrast(img, t_black, t_white):
    '''
    let's make center the percentile of actual intensities -
    removes some of the guesswork.
    '''
    pixels = img.getdata()

    t_black *= 2.55
    t_white *= 2.55

    transformed = []
    denom = float(t_white - t_black)

    for p in pixels:
        q = (p - t_black) / denom
        r = min(max(0, q), 1)
        transformed.append(int(255 * r))

    img.putdata(transformed)


def apply_half_contrast(img, t_black, t_white):
    '''
    apply a less stringent contrast enhancement to the native
    image that will serve as the background for marked[xy].png
    '''
```

```
    diff = int(0.6 * ( t_white - t_black ))
    apply_contrast(img, t_black - diff, t_white + diff)


def mark_pivot(img, t_black, t_white, pivot, suffix):
    '''
    save a copy of the figure with the pivot and the oligomer
    radius marked
    '''
    pixels = img.load()
    by_int = []
    w, h = img.size

    for x in range(w):
        for y in range(h):
            by_int.append((pixels[x,y], x, y))

    # find the 10% brightest pixels ...
    by_int.sort()
    use = len(by_int) / 10

    # ... and then their average distance from the center
    distances = []
    cx, cy = pivot

    for i, x, y in by_int[-use:]:
        distances.append( ((x-cx)**2 + (y-cy)**2)**0.5)

    avg_dist = int(sum(distances) / use)

    # enhance contrast while still in grayscale mode
    apply_half_contrast(img, t_black, t_white)
    rgb = img.convert('RGB')
    paint = ImageDraw.Draw(rgb)

    x,y = img.size
    r = int(max(2, 0.025 * x))
    bbox = (cx - r, cy - r, cx + r, cy + r)

    paint.ellipse(bbox, fill='red')

    # draw circle through peak intensities
    bbox = (cx - avg_dist, cy - avg_dist, cx + avg_dist, cy + avg_dist)
    paint.ellipse(bbox, outline='red')

    rgb.save('marked%s.png' % suffix)
    return avg_dist


def do_n_mer(n, xoff, yoff, t_black, t_white, suffix):
```

```
'''
perform overlay and averaging for a given subunit
stoichiometry (n), with the center of rotation offset
by xoff and yoff. Adjust overlaid picture using contrast
and brightness, and save to file using suffix.
'''
angles = [ 360.0 * i / n for i in range(n) ]

# open image, make sure it is in gray scale mode
native = Image.open(original_file).convert('L')
native_width, native_height = native.size

transformed = []

placement = native_width + xoff, native_height + yoff
pivot = native_width / 2 + xoff, native_height / 2 + yoff

radius = mark_pivot(native, t_black, t_white, pivot, suffix)

for angle in angles:
    canvas = Image.new("RGB", (2 * native_width, 2 * native_height), "red")
    draw_pivot(canvas, native, angle, pivot, placement)
    transformed.append(canvas.load())

# average all images
averaged = Image.new("L", (2 * native_width, 2 * native_height), 0)
pixels_avg = averaged.load()

width, height = canvas.size

red = (255,0,0)

for x in range(width):
    for y in range(height):
        pixels_raw = [ r[x,y] for r in transformed ]

        if red in pixels_raw: # not overwritten in at least one frame
            pixels_avg[x,y] = 0
        else:
            reds = [ p[0] for p in pixels_raw ]
            r = sum(reds) / len(reds)
            pixels_avg[x,y] = r

p0, p1 = pivot
cropped = averaged.crop((p0, p1, p0 + native_width, p1 + native_height))

apply_contrast(cropped, t_black, t_white)
cropped.save('averaged%s%s.png' % (n, suffix))
return radius
```

```
if_name == '_main_':

    import sys,  os
    curdir = os.getcwd()

    os.chdir(sys.argv[1])

    x_center, y_center, span, t_black, t_white = [int(arg) for arg in sys.argv[2:]]

    xr = [x_center - span, x_center, x_center +span]
    yr = [y_center - span, y_center, y_center + span]

    suffixes = "lzh"

    for i  in range(n_from, n_to+1):
        print "doing", i
        if span > 0:
            for j, x in enumerate(xr):
                for k, y in enumerate(yr):
                    suffix = suffixes[j] + suffixes[k]
                    do_n_mer(i, x, y, t_black, t_white, suffix)
        else:
            radius = do_n_mer(i, x_center, y_center, t_black, t_white, "zz")
            print radius

    print "done", os.path.basename(os.getcwd()), "center offsets (x,y):", xr, yr

    # end of program
```

References

[1] D. M. Czajkowsky et al.: Staphylococcal alpha hemolysin can form hexamers in phospholipid bilayers. *J. Mol. Biol.* 276 (1998), 325–30. pmid: 9512705.
