# Supplementary material for: Sialic acid facilitates binding and cytotoxic activity of the pore-forming Clostridium perfringens NetF toxin to host cells
Source: PLoS One. 2018 Nov 7;13(11):e0206815. doi: 10.1371/journal.pone.0206815 (PMC6221314; doi:10.1371/journal.pone.0206815)
Supplement: S2 Fig — Inhibition of rNetF (A) and rDelta (B)-mediated hemolysis by gangliosides. (PDF) [file pone.0206815.s002.pdf]

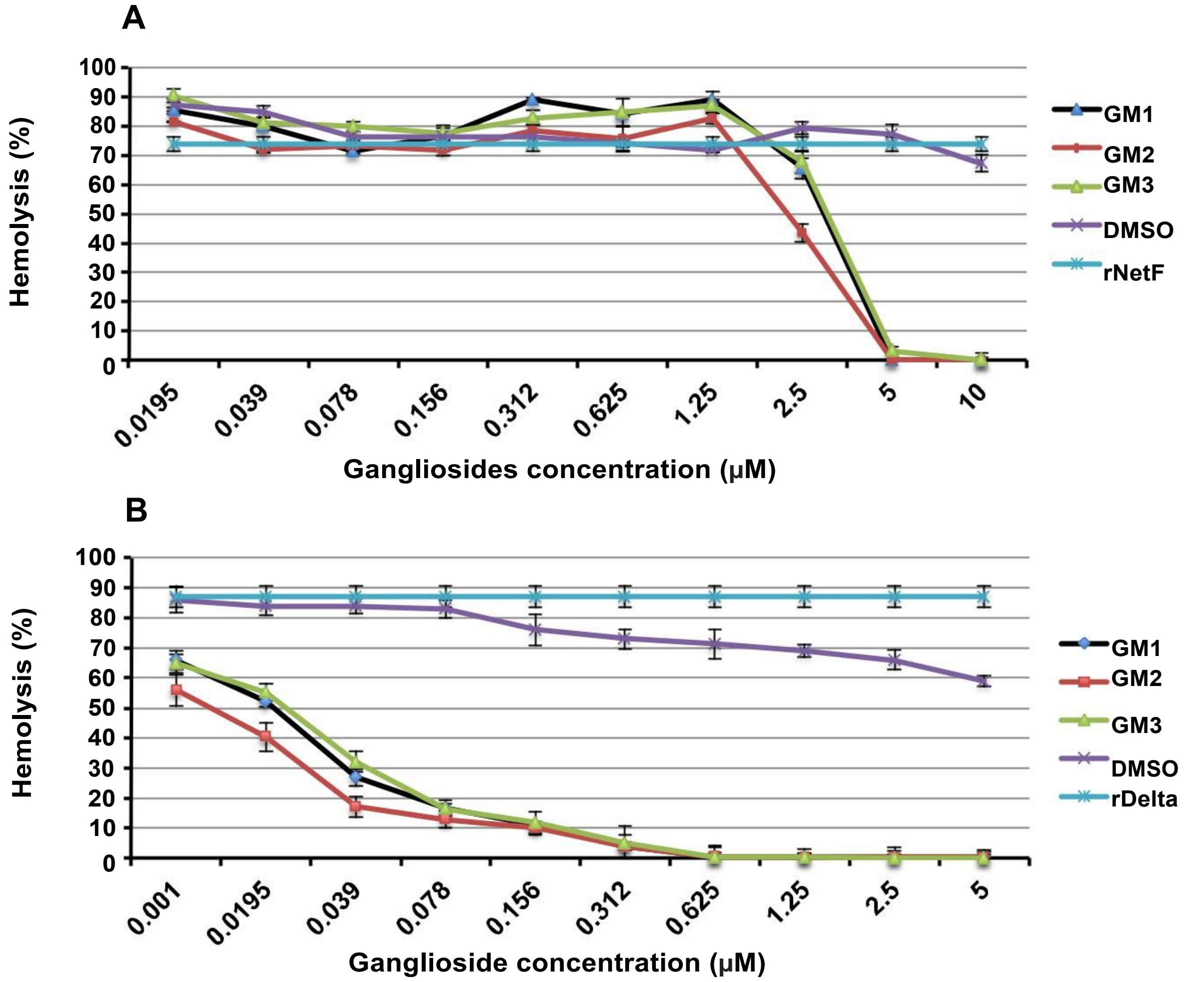

S2 Fig: Inhibition of rNetF (A) and rDelta (B)-mediated hemolysis by gangliosides. Both rNetF (1.3 µg/ml) and rDelta (10 ng/ml) were incubated with increasing concentration of GM1, GM2, and GM3 for 20 min at 37°C. Then, 2.5% sheep RBCs were added and incubated at 37°C for 1 h. Subsequently, the cells were pelleted and the supernatants were removed. Hemolysis was determined by measuring absorbance at 424 nm. PBS was used as the negative control for 0% hemolysis and 2% Triton X-100 as the positive control for 100% hemolysis. The values are averages of three experiments that were carried out in triplicate each; error bars represent the standard deviations across all 9 data points. ANOVA (parametric distribution) and Kruskal-Wallis (non-parametric distribution) tests were used for statistical analysis. The two tests do not detect a significant difference between the curves ($p > 0.05$).
