# Supplementary material for: Sialic acid facilitates binding and cytotoxic activity of the pore-forming Clostridium perfringens NetF toxin to host cells
Source: PLoS One. 2018 Nov 7;13(11):e0206815. doi: 10.1371/journal.pone.0206815 (PMC6221314; doi:10.1371/journal.pone.0206815)
Supplement: S3 Fig — (PDF) [file pone.0206815.s003.pdf]

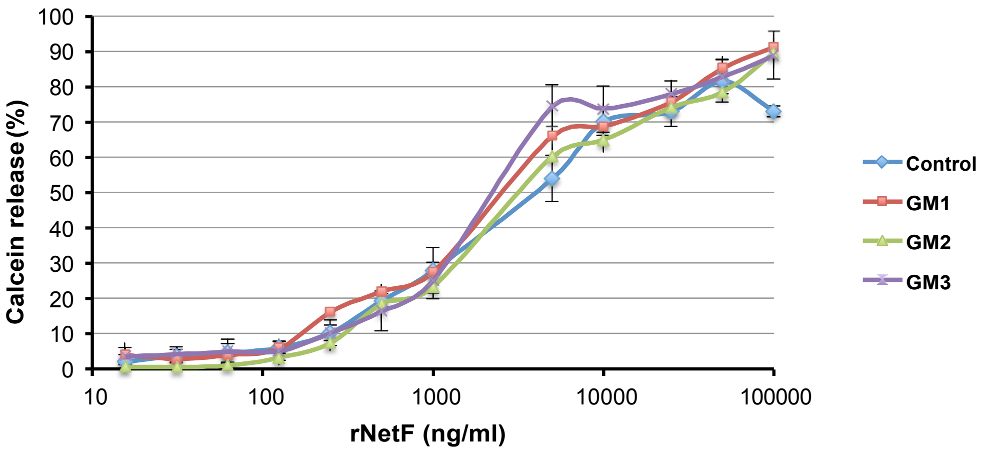

S3 Fig: rNetF-induced calcein release from liposomes containing gangliosides. Liposomes (DOPC: DOPG: Cholesterol) containing GM1, GM2, or GM3 (at 2% of total lipid) were exposed to rNetF at the concentrations indicated for 1h at 25°C, and the release of calcein entrapped within was measured by fluorescence (excitation: 475 nm; emission: 516 nm). 100% release was determined using detergent (Triton X-100). The values are averages of three experiments that were carried out in triplicate each; error bars represent the standard deviations across all 9 data points. ANOVA (parametric distribution) and Kruskal-Wallis (non-parametric distribution) tests were used for statistical analysis. The two tests do not find evidence for a difference between the curves ($p > 0.05$).
