# Supplementary material for: Sialic acid facilitates binding and cytotoxic activity of the pore-forming Clostridium perfringens NetF toxin to host cells
Source: PLoS One. 2018 Nov 7;13(11):e0206815. doi: 10.1371/journal.pone.0206815 (PMC6221314; doi:10.1371/journal.pone.0206815)
Supplement: S4 Fig — (PDF) [file pone.0206815.s004.pdf]

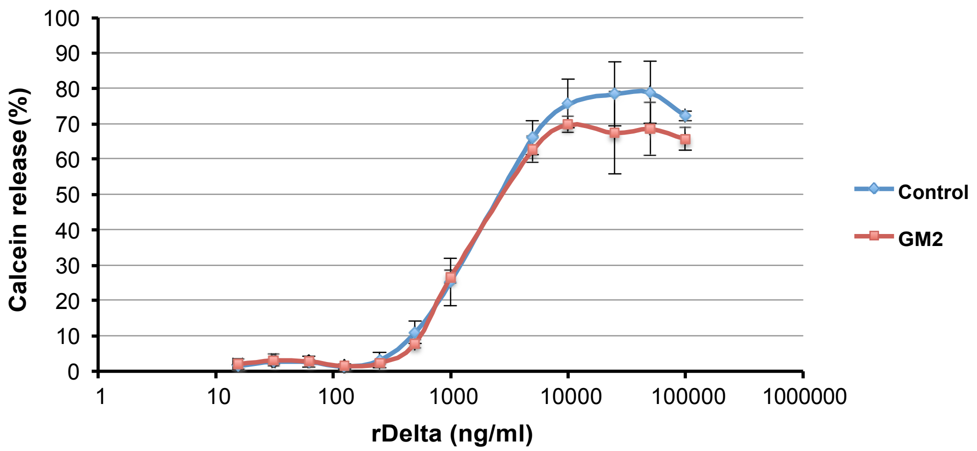

S4 Fig: rDelta induced calcein release from liposome containing GM2. Liposomes with and without GM2 were incubated with different concentrations of rDelta at 25°C for 1 h. The samples were then diluted and calcein fluorescence intensity was measured (excitation: 475 nm; emission: 516 nm). The total calcein release was measured by spectrofluorometer and normalized against its corresponding positive control (0.01% Triton X-100). The values are averages of three experiments that were carried out in triplicate each; error bars represent the standard deviations across all 9 data points. No significant difference was observed between two curves (ANOVA; $p > 0.05$).
